# Incidence, treatment, and outcomes of isoniazid mono-resistant *Mycobacterium tuberculosis* infections in Alberta, Canada from 2007-2017

**DOI:** 10.1371/journal.pone.0229691

**Published:** 2020-03-10

**Authors:** Brett D. Edwards, Jenny Edwards, Ryan Cooper, Dennis Kunimoto, Ranjani Somayaji, Dina Fisher

**Affiliations:** 1 Department of Medicine, University of Calgary, Calgary, Alberta, Canada; 2 Pharmacy Services, Alberta Health Services, Calgary, Alberta, Canada; 3 Division of Infectious Diseases, Department of Medicine, University of Alberta, Edmonton, Alberta, Canada; 4 Department of Microbiology, Immunology, and Infectious Diseases, University of Calgary, Calgary, Alberta, Canada; 5 Department of Community Health Sciences, University of Calgary, Calgary, Canada; McGill University, CANADA

## Abstract

Isoniazid resistant *Mycobacterium tuberculosis* (Hr-TB) is the most frequently encountered TB resistance phenotype in North America but limited data exist on the effectiveness of current therapeutic regimens. Ineffective treatment of Hr-TB increases patient relapse and anti-mycobacterial resistance, specifically MDR-TB. We undertook a multi-centre, retrospective review of culture-positive Hr-TB patients in Alberta, Canada (2007–2017). We assessed incidence and treatment outcomes, with a focus on fluoroquinolone (FQ)-containing regimens, to understand the risk of unsuccessful outcomes. Rates of Hr-TB were determined using the mid-year provincial population and odds of unsuccessful treatment was calculated using a Fisher’s Exact test. One hundred eight patients of median age 37 years (IQR: 26–50) were identified with Hr-TB (6.3%), 98 of whom were able to be analyzed. Seven percent reported prior treatment. Rate of foreign birth was high (95%), but continent of origin did not predict Hr-TB (p = 0.47). Mean compliance was 95% with no difference between FQ and non-FQ regimens (p = 1.00). Treatment success was high (91.8%). FQ-containing regimens were frequently initiated (70%), with no difference in unsuccessful outcomes compared to non-FQ-containing regimens (5.8% vs. 13.8%, OR 0.4, 95% CI 0.1–2.3, p = 0.23). Only one patient (1%) utilizing a less common non-FQ-based regimen including two months of pyrazinamide developed secondary multidrug resistance. Unsuccessful treatment was low (<10%) relative to comparable literature (~15%) and showed similar outcomes for FQ and non-FQ-based regimens and no deficit to those using intermittent fluoroquinolones in the continuation phase of treatment. Our findings are similar to recent data, however prospective, randomized trials of adequate power are needed to determine the optimal treatment for Hr-TB.

## Introduction

Infection with *Mycobacterium tuberculosis* (TB) has afflicted humans for millennia[1] and it is estimated that at least 25% of the current worldwide community is infected with TB. TB remains the highest infectious cause of death worldwide with more than 10 million new cases and nearly two million deaths in 2016[2,3].

Treatment of active TB remains long and arduous, particularly in the setting of drug resistance. Unfortunately, the worldwide proportion of drug-resistant TB infections continues to increase[4,5], due in part to impaired access to drug-susceptibility testing in some settings and subsequent first-line treatment of unidentified resistance, poor access to alternative drug regimens, and worldwide challenges in adherence[3].

The standard WHO treatment for active TB recommendation comprises two months of isoniazid, rifampin, pyrazinamide, and ethambutol (HRZE), followed by four months of INH and rifampin (HR)[6]. Isoniazid is considered a backbone of therapy because of its high early bactericidal activity combined with a good safety profile[7]. Unfortunately, drug resistance to isoniazid has become a global concern and inappropriate treatment of unidentified isoniazid mono-resistance can lead to further resistance to the other core anti-TB agent, rifampin, in an estimated 8% of new cases[4,8].

The prevalence of drug resistance in Canada has remained relatively stable over the past decade. In Canada, the most commonly encountered TB resistance profile is isoniazid-resistance (Hr-TB)[5,9]. In 2016, 9% of all isolates in Canada demonstrated any resistance (compared with 17% worldwide), with 83% of these demonstrating mono-resistance (overall approximately 6% were Hr-TB). This is lower than the worldwide estimates of Hr-TB at 8–10%[2,10]. However, through *in vitro* and phylogenetic studies, it has been suggested that unrecognized isoniazid mono-resistance worldwide may be contributing substantially to the MDR epidemic[8] with selective antimicrobial pressure prompting rifampin-resistant proliferation[11,12].

Until recently, there has been a paucity of data to guide best treatment for Hr-TB, which is associated with worse outcomes than fully-sensitive TB[13,14]. The need remains for data from randomized controlled trials. Treatment success of Hr-TB is roughly 80–85%[8,15–17] compared with 90–95% for drug-susceptible disease[18]. As such, we sought to review the epidemiology, incidence, and outcomes of various treatment regimens over 11 years in a multi-centre, low incidence, western Canadian region. Consistent with recent 2019 World Health Organization TB treatment guidelines for Hr-TB[19], we explored regimens including fluoroquinolones (FQ) and those extending the duration of pyrazinamide to at least 6 months with particular interest, to help inform our clinicians locally and in other low incidence settings.

## Materials and methods

### Population

All individuals with suspected or confirmed TB infection have care coordinated through one of three centralized comprehensive clinics in the province of Alberta. A clinic is located in each of the two major urban centres (Edmonton and Calgary) and a third clinic provides care coordinated from Edmonton but delivered locally to the remaining smaller mostly rural centres. Treatment is individualized by clinicians through the regional clinic to which each patient belongs. This often elicits regional differences in treatment practice. For example, patients treated in the Edmonton region are more frequently initiated on intermittent thrice weekly FQ dosing in the continuation phase based on local experience than in the Calgary region. Consequently, in Calgary, pyrazinamide is more commonly discontinued after two months. Intermittent, thrice weekly continuation phase dosing for more commonly utilized medications including isoniazid and rifampin is more frequent across all sites and all treatment is directly observed.

The clinics offer significant treatment support. Patients are intended to have physician follow-up on a minimum bimonthly basis. Patients are assigned a nursing case worker who assists in coordinating directly observed therapy (at the clinic or participating pharmacies) and laboratory collections, are available to discuss patient concerns including adherence or toleration concerns and may alert physicians to the need for more frequent patient follow-up if indicated. TB outreach nurses visit patients in their homes when there are challenges accessing the clinic, adherence issues, or other social issues that necessitate visits. Clinics also have available onsite pharmacist and social worker support.

We undertook a retrospective cohort study of all patients identified in the province of Alberta with bacteriologically confirmed, culture-positive TB with Hr-TB susceptibility pattern from January 1, 2007 –December 31, 2017. Hr-TB was defined as isolates with phenotypic resistance to isoniazid among usual first-line anti-mycobacterial agents (isolates were sensitive to rifampin, pyrazinamide, and ethambutol). The incident positive isolate determined the year of infection.

We included all patients, regardless of age and included both pulmonary and extra-pulmonary cases of TB. Patients were excluded if they had insufficient clinical data to determine treatment or outcome parameters.

Patients were identified using iPHIS (integrated Public Health Information System). This Public Health database is used by Alberta Tuberculosis Services and tracks patient demographics, culture results and susceptibilities, treatment regimens, and treatment outcomes, including requirement for retreatment. All demographic, treatment, and outcome data is available from this database and it also includes electronic multi-disciplinary progress notes. All patient data was collected and subsequently anonymized by removing names, date of birth, and personal health numbers and replaced with a unique study identification number. This was stored on a study key. If patient information was missing, it was supplemented by referring back to the electronic record by linking the study identification number to the original patient. All dates (treatment, immigration, hospitalization) were adjusted by a randomly selected number to preserve the anonymity of participants. Adverse events were collected by physician report documented in the database. Where possible, we linked reported adverse events with objective measures (elevated ALT for hepatotoxicity), but this was not always documented in the electronic record.

### Regimens

Patients were categorized by treatment regimen according to the composition of the initial anti-mycobacterial regimen selected once Hr-TB was identified. Therefore, if a patient’s regimen included a FQ once isoniazid resistance was recognized by the clinician, they were categorized as utilizing a FQ-containing regimen. Those who started with a non-FQ-containing regimen, but later had a fluoroquinolone added (to replace another medication for toxicity) were denoted as such. While initial intended duration of an anti-mycobacterial (e.g. pyrazinamide) was not always apparent, we denoted those that continued pyrazinamide to at least six months, while collecting available data on reasoning to discontinue it earlier (e.g. stopped at two months per protocol versus discontinued early for intolerance).

### Outcomes

The primary outcome was the odds of unsuccessful treatment, a composite of (i) treatment failure, (ii) relapse, (iii) new resistance acquisition, (iv) incomplete treatment, and (v) death, based on the regimen used for Hr-TB. Treatment failure and relapse mirror those defined by WHO for non-MDR-TB[20], while incomplete treatment was invoked to include patients who did not complete the prescribed duration of therapy but continued to follow up with the clinics. Other outcomes analyzed included annual incidence of Hr-TB during the study period and risk of Hr-TB by country of origin.

Data was analyzed using *R* and STATA[21,22]. Patient demographics were summarized. Rates of resistance were calculated by the number of cases divided by the mid-year population of the province using census data[23]. Odds of unsuccessful treatment by regimen were determined using a Fisher’s exact test. Confidence intervals for treatment success were calculated using a Wald proportion CI calculator. Two-tailed significance was set at p<0.05. Ethics approval of the study was obtained through the Conjoint Health Research Ethics Board (REB18-1673). Need for informed consent of the study participants was waived due to the retrospective nature of the study dating back 11 years and the impracticality of obtaining consent in those circumstances.

## Results

### Patients

**Fig 1:**
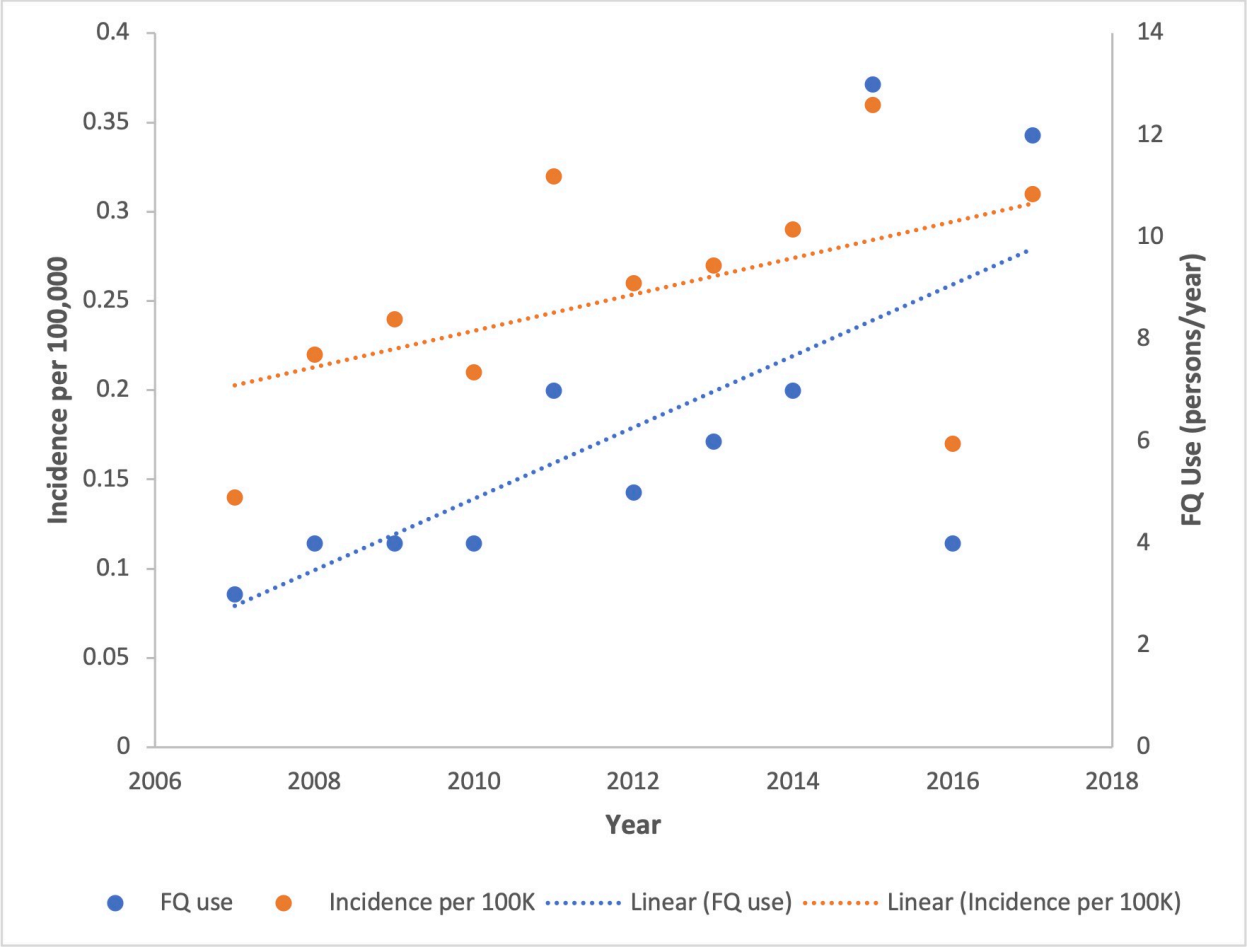
Incidence of Hr-TB per 100,000 per year and inclusion of fluoroquinolones in initial Hr-TB regimens per year.

We identified 108/1718 (6.3%) patients in our population with Hr-TB in the eleven-year study period. The mean annual incidence rate was 0.25/100,000 (SD: 0.06) (Fig 1). We do not have outcome data for eight patients who transferred to a new jurisdiction and two patients died prior to treatment initiation, leaving 98 patients available for analysis. Ofloxacin susceptibility was confirmed for 95/98 patients (96.9%). The median age of the cohort was 37 years (IQR: 26–50) and consisted of 53.1% females. Eighty-four percent of the cohort was living within the two large urban centres in Alberta (Calgary: 42; Edmonton: 40 patients). The proportion of pediatric TB, occurring in patients <15 years of age, was 1/98 (1.0%). Six patients (6.1%) were HIV (+). There were 7/98 Hr-TB patients (7.1%) with previous TB treatment reported prior to the study. These patients had a mean age of 50 years, all were foreign-born, and all were negative for HIV.

**Fig 2:**
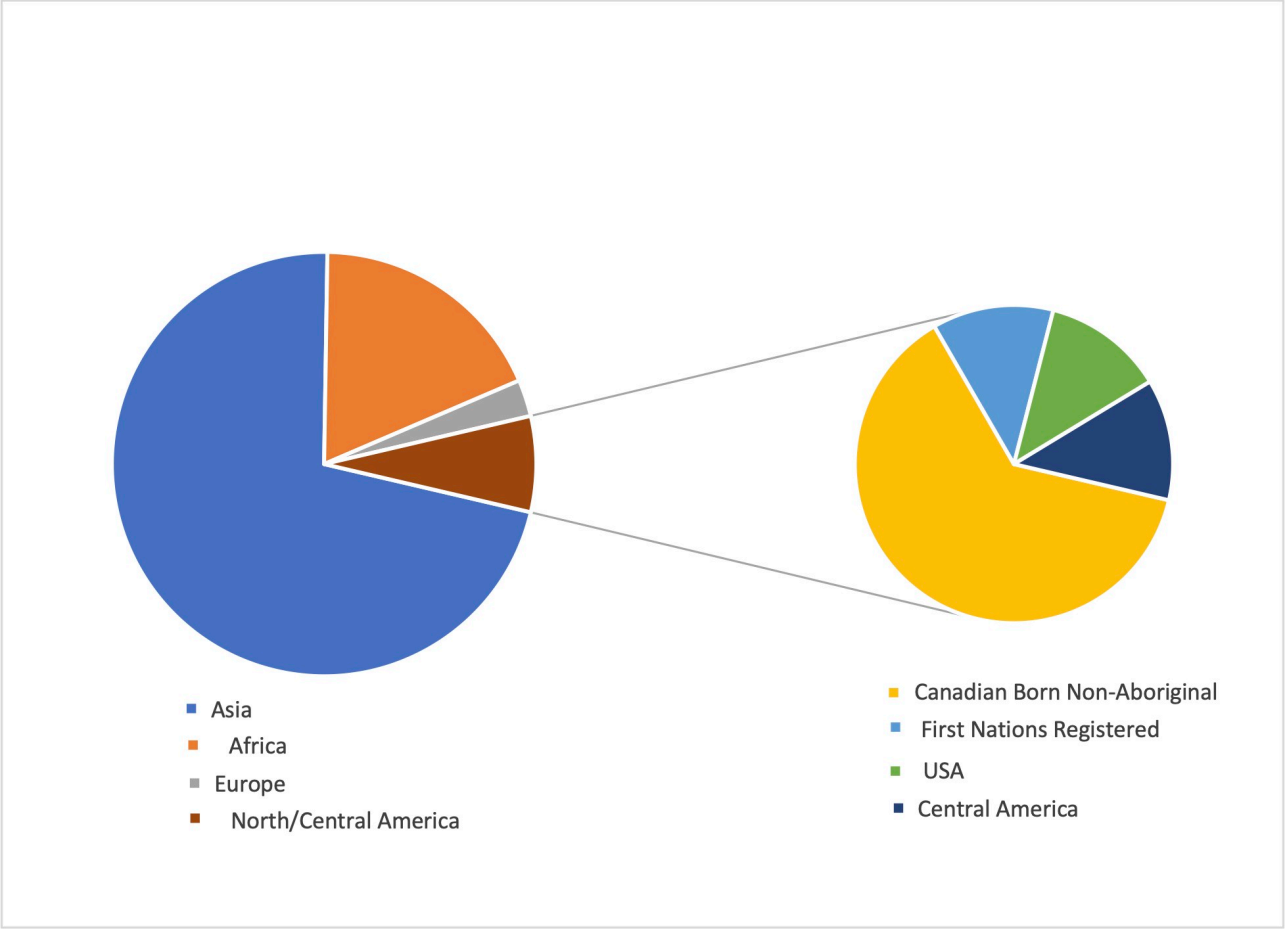
Distribution of Hr-TB by region and ethnicity.

The distribution of country of origin was broad with 93 patients (95%) born outside of Canada. (Fig 2). There was no difference in incidence of Hr-TB by region: Asia 69/998 (6.9%), Europe 3/44 (6.8%), and Africa 19/352 (5.4%), p = 0.47. The median time to diagnosis of active tuberculosis after immigration was 5.2 years (IQR: 1.3–12.1).

### Site of disease

Pulmonary infection was confirmed in 60 patients (61%). Extra-thoracic lymphadenitis was the most common site of extrapulmonary disease, affected in 25 patients (25%). Twelve patients (12%) had pleural disease, seven patients (7.1%) had gastrointestinal disease and seven had musculoskeletal involvement, while two (1.8%) had culture-proven disease involving the genitourinary system. There was one patient with disease involving the central nervous system and one involving the pericardium.

### Regimens

Sixty-nine patients (70%) were initiated on a regimen that included a FQ. An additional six patients later had one added to their regimen to replace a discontinued anti-mycobacterial. Thirty-eight of these patients (51%) used an intermittent regimen (3 days per week) of fluoroquinolone during the continuation phase. The regimens utilized are outlined in Table 1. The mean age did not differ between those started on FQ-containing regimens (40.2) versus not (39.9), p = 0.94. Nine patients, median age 27 years (IQR: 17–38), utilized regimens with pyrazinamide continued for 6 months [median 9 months (IQR: 6.6–9.1), 4/9 of these on FQ-containing regimens], with mean compliance 93.2%. Thirty-one patients had isolates that lacked high-level isoniazid resistance (concentration >1mg/L). The mean compliance across all patients and regimens analyzed was 95% and did not differ between regimens including fluoroquinolones (95.0%) and those not (95.0%), p = 1.00. Thirty-one patients (32%) experienced adverse events that prompted treatment alteration. Nine patients (9%) experienced elevated liver enzymes/hepatoxicity, with six of these attributed to pyrazinamide, which abbreviated use to a maximum of 1 month. The remainder of toxicity was composed of a distribution of gastrointestinal, cutaneous, hematologic, neurologic, and visual signs and symptoms (Table 2).

**Table 1:** Regimens utilized for Hr-TB and outcomes observed.

Regimen Initiated	Number (n = 98)	Median Age (IQR)	Duration of Z* (# Stopped for Toxicity)	FQ Later Added (n = 6)	FQ 3xW Used** (n = 38)	Adverse Event(s) Prompting Treatment Change	Duration Of Treatment	Outcome
**Fluoroquinolone Included in Initial Regimen (n = 69)**								
(H)REZFQ	36	36.1 (29.9–43.5)	<2 months: 5 At 2 Months: 26 >2 to <6 Months: 1 (At Death) ≥6 Months: 4 (For Toxicity: 7)	-	17	17	6 Months: 6 >6 Months: 28 Incomplete: 2	Treatment Completion: 22 Cure: 12 Died: 1 Non-Compliant: 1
(H)REFQ	10	50.8 (38.9–66.6)	-	-	5	4	>6 Months: 9 Incomplete: 1	Treatment Completion: 5 Cure: 4 Died: 1
(H)RZFQ	22	37.6 (24.9–49.5)	<2 Months: 1 (At Death) At 2 Months: 18 >2 to <6 Months: 3 ≥6 Months: 0 (For Toxicity: 0)	-	14	2	6 Months: 4 >6 Months: 17 Incomplete: 1	Treatment Completion: 12 Cure: 9 Died: 1
Other Regimen Including FQ (12R+FQ+LZD)	1	15.4	-	-	0	0	>6 Months: 1	Cure: 1
**Fluoroquinolone Not Included in Initial Regimen (n = 29)**								
(H)REZ	28	33.3 (25.1–55.6)	<2 Months: 3 At 2 Months: 19 >2 to <6 Months: 1 ≥6 Months: 5 (For Toxicity: 3)	To Replace Another Drug: 6	2	7	6 Months: 8 >6 Months: 18 Incomplete: 2	Treatment Completion: 21 Cure: 3 Died: 1 Non-Compliant: 2 Relapse with new MDR-TB: 1
(H)RE	1	33.8	-	0	0	1	>6 months: 1	Cure: 1

H: isoniazid; R: Rifamycin; Z: Pyrazinamide; E: Ethambutol; FQ: Fluoroquinolone (moxifloxacin and/or levofloxacin); LZD: Linezolid.

*Those not stopped for toxicity were stopped at the discretion of the treating clinician for perceived adequate treatment.

**Use of FQ used 3x Weekly in Continuation Phase.

**Table 2:** Adverse effects prompting treatment change, categorized by regimen.

Regimen Initiated	Adverse Event Prompting Treatment Change (%)	Z Stopped Early for Hepatoxicity (Duration)	Z Continued for ≥6 Months	Other Adverse Events
(H)REZ (n = 28)	7 (25)	2 (<1 month)	5	Neuropathy (n = 2) GI (n = 1) Thrombocytopenia (n = 1) Dizziness (n = 1)
(H)RE (n = 1)	1 (100)	-	-	Hypersensitivity Reaction (n = 1)
(H)REZFQ (n = 36)	17 (47)	3 (≤1 month)	4	Thrombocytopenia (n = 1) Cutaneous (n = 3) Visual (n = 2) GI (n = 4) Tendinopathy (n = 3) Hepatotoxicity (n = 1) Arthralgia (n = 1)
(H)REFQ (n = 10)	4 (40)	1 (<1 month)	-	GI (n = 1) Arthralgia (n = 2)
(H)RZFQ (n = 22)	2 (9)	0	0	Thrombocytopenia (n = 1) GI (n = 1)
12R + FQ + LZD (n = 1)	0 (0)	-	-	-

H: isoniazid; R: Rifamycin; Z: Pyrazinamide; E: Ethambutol; FQ: Fluoroquinolone (moxifloxacin and/or levofloxacin); LZD: Linezolid.

### Treatment outcome

Treatment success occurred in 90/98 patients (91.8%, 95% CI 86.4–97.3%) (60 treatment completion, 30 cure). Four patients died during treatment. Four patients had incomplete treatment, discontinuing therapy prior to planned completion.

After excluding patients who transferred to a new jurisdiction, treatment success was high in both patients using regimens with fluoroquinolones 65/69 patients (94%) and those without 25/29 (86%) (Table 1). Odds of unsuccessful treatment was no different between the two groups (4/69, 5.8%) vs. not (4/29, 13.8%), OR 0.4, 95% CI 0.1–2.3, p = 0.23 (Table 3). Nineteen of 29 patients (66%) in the non-FQ containing group had prolonged therapy (i.e. >6 months). Of these, 18/19 (95%) were successful with one developing new resistance. Of the non-FQ group, five received pyrazinamide for ≥6 months with none experiencing unsuccessful outcomes. In the FQ group, there were no detected cases of treatment *relapse*, *failure*, or new acquired resistance development. Intermittent FQ use was broadly distributed in approximately 50% of each of the FQ-containing regimens. Low rates of unsuccessful treatment were observed (2/38, 5.3%) (both due to non-compliance) which was no higher than those on daily regimens (5/34, 14.7%; p = 0.17). Of the nine patients using pyrazinamide ≥6 months, there were no unsuccessful outcomes and none experienced hepatotoxicity, although two stopped treatment between month 6 and 7 due to rash and GI symptoms. Overall, ten patients were forced to stop pyrazinamide earlier than planned, three of whom had unsuccessful outcomes (1 death, 2 incomplete treatment) (Table 3).

**Table 3:** Patients with unsuccessful outcomes.

Patient (n = 8)	Initial Regimen	Medication Durations and Reasons for Discontinuation (if applicable)	Outcome
**Fluoroquinolone Included in Initial Regimen**			
30–35 y.o. F HIV (+) Disseminated TB	(H)REZFQ	E: 2 months R: 3.6 months Z: 3 months Moxifloxacin/Levofloxacin: 3.6 months	Died
80–85 y.o. M Pulmonary TB	(H)RZFQ	R: 1.5 months Z: 1.5 months Moxifloxacin: 1.5 months	Died
25–30 y.o. F Lymphadenitis	(H)REZFQ	E: 8.5 months R: 8.5 months Z: 2.1 months (stopped at 2-month assessment) Levofloxacin: 1.9 months	Non-compliant; No relapse ≥6 months after discontinuation
60–65 y.o. M Pulmonary TB	(H)REFQ	E: 5.9 months R: 5.9 months Levofloxacin: 5.9 months	Died from pre-existing condition
**Fluoroquinolone Not Included in Initial Regimen**			
75–80 y.o. M Pulmonary/lymphadenitis/ (presumed) vertebral TB	(H)REZ	E: 10.9 months R: 10.9 months Z: 2 months (stopped at 2-month assessment) Regimen discontinued for thrombocytopenia	Adverse effects (thrombocytopenia) with incomplete treatment. Development of MDR-TB
80–85 y.o. M Pulmonary and CNS (presumed) TB	(H)REZ	E: 2.4 months R: 2.0 months Z: 0.4 months (stopped for hepatotoxicity) Levofloxacin/Moxifloxacin: 2.4 months (added to replace Z)	Died
55–60 y.o F Pulmonary TB	(H)REZ	E: 8.7 months R: 7.6 months Z: 0.9 months (stopped for hepatotoxicity) Levofloxacin: 1.7 months (temporary replacement for Z; ultimately continued on RE)	Adverse effects (hepatotoxicity) and non-compliance; No relapse ≥18 months after discontinuation
40–45 y.o. F Pulmonary TB	(H)REZ	E: 1.9 months R: 3 months Z: 0.7 months (stopped for GI) Levofloxacin: 2.3 months (Added to replace Z)	Adverse effects (GI) and non-compliance; No relapse ≥6 months after discontinuation

H: Isoniazid; E: Ethambutol; R: Rifampin; Z: Pyrazinamide; FQ: Fluoroquinolone (Moxifloxacin or Levofloxacin).

MDR-TB: Multi-drug Resistant Tuberculosis.

We assessed the individual components of the primary outcome. After excluding those who died, incomplete treatment was no different between FQ-containing and non-FQ-containing regimens: 1/66 vs. 2/28, p = 0.15. Similarly, death was no different between the two groups: 3/69 vs. 1/29, p = 0.83. Three of the four patients who died on treatment were over the age of 60.

Development of secondary resistance after treatment was low (1/98, 1.0%). One patient developed multi-drug resistant tuberculosis eighteen months after treatment. This 77-year-old male patient was initially diagnosed as disseminated disease (pulmonary, lymphadenitis, and vertebral) and treated with a fluoroquinolone-free regimen comprised of 2 months of pyrazinamide plus 11 months of rifampin/ethambutol with ≥94% compliance. This uncommon regimen was chosen due to his comorbidity and is included in the Canadian guidelines[4]. Therapy was stopped at 11 months (1 month early) due to adverse effects. He developed recurrent disease at the vertebral site with MDR-TB requiring prolonged therapy.

Of the 31 patients with confirmed isolates lacking high level isoniazid resistance, seven were treated with regimens including isoniazid for a minimum of two months and all had treatment success. Of the 24 not treated with isoniazid, 20 (83%) were successfully treated; p = 0.25.

Specific to patients exclusively with extrapulmonary TB, there was no difference in treatment success between those with FQ-based regimens versus those without: 25/27 (92.6%) vs. 11/11 (100%); p = 0.35.

## Discussion

In our longitudinal cohort study, we identified an incidence of Hr-TB consistent with rates across Canada and lower than estimated global rates[9]. While low overall, the incidence appears to be increasing (Fig 1). Identified risk factors for Hr-TB include foreign-born status and prior TB treatment[24]. Foreign-born status was very frequent in our cohort, with the highest rates of Hr-TB occurring in patients originally from Asian countries. Half of these cases were identified within five years of immigration. No other risk factors predicting Hr-TB were identified.

Treatment of Hr-TB is associated with worse outcomes than is treatment of fully-sensitive TB[14]. A recent meta-analysis highlighted high levels of treatment failure, relapse, and MDR acquisition (11%, 10%, and 8% respectively) in Hr-TB patients treated with standard ‘new’ first line drug therapy and 6%, 5%, and 3% in those treated with the *retreatment* regimen previously endorsed by WHO[8]. Despite these findings, there has yet to emerge a clear consensus on the best treatment for Hr-TB, including the duration and the need for fluoroquinolone inclusion in the regimen[13,14], and there are no randomized trials. Commonly encountered regimens include rifampin, ethambutol, and pyrazinamide (REZ) for 6 months or longer, or 2 months of pyrazinamide given with at least 6 months of fluoroquinolone, rifampin, and ethambutol. A subsequent, patient-level meta-analysis of nearly 4000 patients found that extending the REZ duration longer than 6 months had no benefit[14]. There was significant heterogeneity amongst included studies and often with individualized treatment regimen adaptations (possibly for patient or centre characteristics), which was similar to what we found in our multicentre study.

We found success rates of 92% (95% CI: 86%-97%) for all treatments, which is higher than comparable literature of 85%[8,15–17]. It is also important to recognize that in the three patients non-adherent with therapy, the prescribed duration was based on the recommendation of the treating clinician guided by available evidence at the time of care. Only one of these patients had treatment less than 6 months and none have relapsed to date. Therefore, it is arguable that success was higher. The reason for high success rates is likely multifaceted but includes routine access to drug-susceptibility testing and significant allied health treatment support from nursing, pharmacy, social work, and community outreach in our low incidence setting. Directly Observed Therapy is mandated in our province, physician follow-up is scheduled at the previous appointment, and intensity of nursing follow-up is increased with dwindling compliance, including home visits and medication home delivery. The most common non-FQ-based regimen utilized was combined/extended (H)RE with two months of pyrazinamide and had successful results (95%).

Six months (or more) of REZ was relatively uncommon but very successful (100%), however due to the retrospective nature of our data it is impossible to know if this was attempted more but failed due to intolerance. Approximately 10% of our cohort had abbreviated pyrazinamide duration, with six patients (6%) due to hepatotoxicity. While this is on the higher end of previous reports[25,26], the lack of available information on the degree of injury combined with retrospective data, limit the ability to analyze this further. Two of these patients were over the age of 50, three were female, none were HIV (+), and none were documented as using excessive alcohol, all of which have previously been identified as risk factors for hepatotoxicity[25]. Nonetheless, recent US guidelines suggest that in some circumstances pyrazinamide duration may be limited to two months if a fluoroquinolone is used throughout treatment[27]. This regimen was highly successful in our cohort (100%) but generally prompted extension of treatment beyond six months (73%), in line with contemporary Canadian guidelines[4].

Support for the inclusion of fluoroquinolones in TB treatment continues to grow, particularly in regimens where isoniazid cannot be included[28–30]. Most compelling is the above individual patient-level data meta-analysis, which found adding a fluoroquinolone to the REZ combination for 6 months was associated with almost three times higher odds of treatment success in Hr-TB[14]. While our study did not show evidence of higher treatment success, similar to other studies[31,32], this must be considered in light of the limitations of our data. The small sample size impairs our ability to identify meaningful differences based on regimen utilized. Additionally, due to low outcome incidence, we did not compute multivariate analyses. Due to the observational nature of the study, it remains possible that treating physicians were more likely to add a fluoroquinolone for severe cases of HR-TB, thus introducing confounding. Similarly, increased age is a risk factor for worse outcomes and fluoroquinolones are often initiated in patients of advanced age where pyrazinamide is abbreviated or deferred due to concerns of toxicity. There was evidence of this with an older median age in patients initiated on (H)REFQ. There were no outcomes of *failure* or new resistance acquisition detected in this group, contrary to the one case of MDR-acquisition in a regimen that did not include a fluoroquinolone.

There was significant use of both levofloxacin and moxifloxacin in our cohort, with many patients using both during their course of treatment, inhibiting comparison. Inclusion of fluoroquinolones locally increased over the eleven-year span of our study (Fig 1), presumably in association with the growing body of evidence supporting their use, the relative simplicity of administration, and accessibility. Intermittent use of fluoroquinolones during the continuation phase is not common due to a paucity of data. Over half of FQ users utilized intermittent dosing and this was not predictive of poor outcomes in our study. This should be further explored in future studies with standardized intensive phase regimens.

While fluoroquinolones have found their place in the management of drug-resistant TB including Hr-TB, caution must always be exercised. The broad use of fluoroquinolones in the treatment of many bacterial/mycobacterial infections has fostered resistance[33]. Of particular mention is empiric use of fluoroquinolones in community-acquired pneumonia, where TB has not been excluded. Additionally, there is a growing understanding of the potential risks fluoroquinolones may pose in addition to long-quoted risk of tendinopathies, including dysglycemia, QTc prolongation, CNS toxicity, neuropathies, aortic aneurysm rupture, and valvular insufficiency[34–36].

Additional limitations of our study introduced by the retrospective observational design, include the possibility of confounding by indication and underestimation of adverse effects incidences. Nonetheless, the descriptive nature is beneficial to inform our clinicians of recent practices, particularly in a multi-centre study, and compare to most up to date recommendations. The eleven-year time span risks bias from differential time of treatment but captures a greater impression of treatment trends. Finally, it remains plausible that development of further drug resistance will surface in patients treated near the end of our study, underestimating that outcome.

## Conclusion

In this multi-centre study, we found an incidence of Hr-TB of 6.3% with most cases (95%) occurring in foreign-born individuals. There was significant variability in regimens utilized for Hr-TB, consistent with a lack of consensus around best treatment for this pattern of resistance. We found low rates (<10%) of unsuccessful treatment compared to what has previously been reported, and this was regardless of the duration of pyrazinamide, whether fluoroquinolones were included, or whether fluoroquinolones were used intermittently in the continuation phase, all of which is likely a result of the intense treatment support available in our clinics. The only identified case of MDR-acquisition was in a patient whose regimen did not include a fluoroquinolone. These findings are consistent with a recent large meta-analysis. Randomized trials are required to clarify the best treatment for Hr-TB and should consider exploring further the possibility of intermittent dosing of fluoroquinolones in the continuation phase of treatment.
